# From Regulation to Application: The Role of Abscisic Acid in Seed and Fruit Development and Agronomic Production Strategies

**DOI:** 10.3390/ijms252212024

**Published:** 2024-11-08

**Authors:** Xunan Zheng, Weiliang Mo, Zecheng Zuo, Qingchi Shi, Xiaoyu Chen, Xuelai Zhao, Junyou Han

**Affiliations:** 1 Jilin Province Engineering Laboratory of Plant Genetic Improvement, College of Plant Science, Jilin University, Changchun 130062, China; zhengxn21@mails.jlu.edu.cn (X.Z.); mowl@jlu.edu.cn (W.M.); zuozhecheng@jlu.edu.cn (Z.Z.); shiqc23@mails.jlu.edu.cn (Q.S.); xuelai24@mails.jlu.edu.cn (X.Z.); 2 Guangxi Key Laboratory of Animal Breeding, Disease Control and Prevention, College of Animal Science and Technology, Guangxi University, Nanning 530004, China; xychen@st.gxu.edu.cn

**Keywords:** ABA, seed development, fruit development, molecular mechanisms, production applications

## Abstract

Abscisic acid (ABA) is a crucial plant hormone that plays a decisive role in regulating seed and fruit development and is becoming increasingly important in agricultural applications. This article delves into ABA’s regulatory functions in plant growth, particularly during the stages of seed and fruit development. In the seed phase, elevated ABA levels help maintain seed dormancy, aiding seed survival under unfavorable conditions. During fruit development, ABA regulates pigment synthesis and sugar accumulation, influencing the nutritional value and market quality of the fruit. This article highlights three main strategies for applying ABA in agricultural production: the use of ABA analogs, the development of ABA signal modulators, and breeding techniques based on ABA signaling. ABA analogs can mimic the natural functions of ABA, while ABA signal modulators, including enhancers and inhibitors, are used to finely tune plant responses to ABA, optimizing crop performance under specific growth conditions. Furthermore, breeding strategies based on ABA signaling aim to select crop varieties that effectively utilize ABA pathways through genetic engineering and other technologies. ABA is not only a key regulator of plant growth and development but also holds great potential for modern agricultural practices.

## 1. Introduction

**Figure 1:**
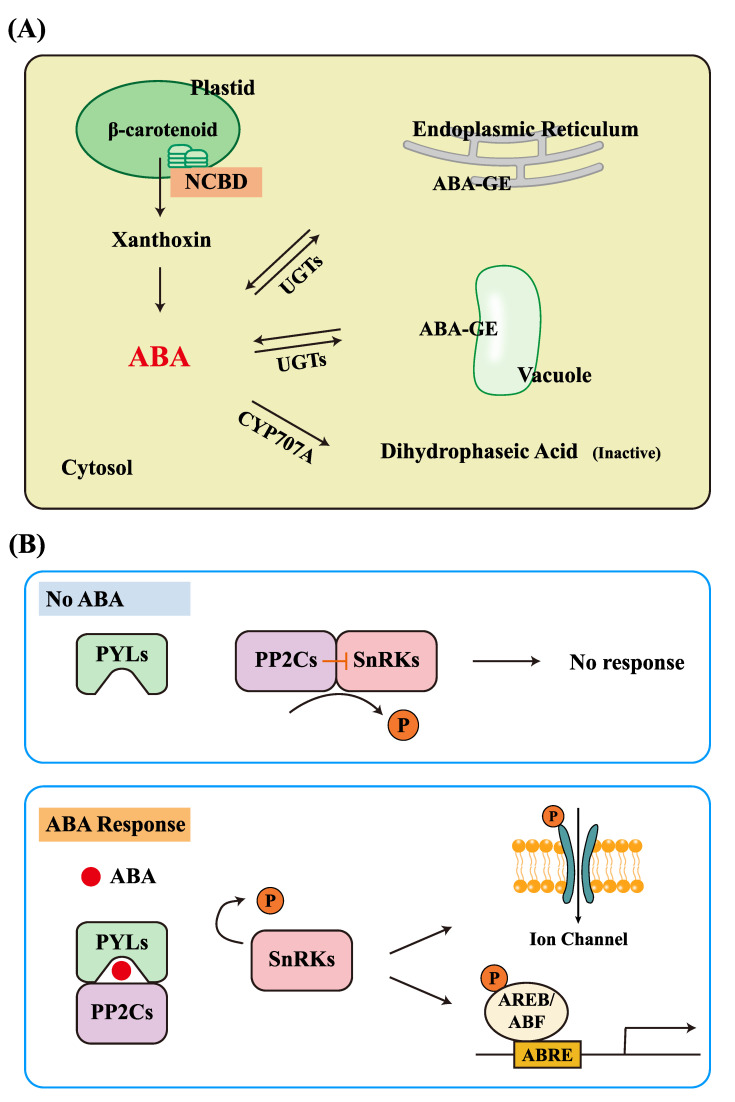
Schematic representation of ABA biosynthesis, metabolism, and signal transduction pathways. (**A**) ABA biosynthesis mainly occurs through the carotenoid pathway, starting with carotenoid synthesis in plastids, particularly the cleavage of β-carotene. NCED is a key rate-limiting enzyme responsible for converting carotenoids into xanthoxin, which is then continuously oxidized in the cytoplasm to form ABA. ABA metabolism primarily follows two pathways: hydroxylation and glycosylation. In the hydroxylation pathway, CYP707A catalyzes the 8′-hydroxylation of ABA, ultimately producing the biologically inactive dihydrophaseic acid. In the glycosylation pathway, ABA can undergo conjugation with glucose, catalyzed by ABA-uridine diphosphate (UDP)glucosyltransferases (UGTs), to form ABA-glucosyl ester (ABA-GE), a biologically inactive storage or transport form of ABA. This inactive form can later be reactivated through enzymatic hydrolysis to release active ABA. (**B**) ABA signal transduction operates through a core module composed of PYL receptors, PP2C phosphatases, and SnRK2 protein kinases. When ABA levels increase, ABA binds to PYL receptors, inhibiting the activity of PP2C phosphatases and releasing the suppression of SnRK2s, thereby activating them. The activated SnRK2s phosphorylate downstream AREB/ABF transcription factors, which bind to ABRE cis-acting elements in DNA to activate the expression of target genes. Additionally, SnRK2s can activate certain ion channels, promoting stomatal closure.

Abscisic acid (ABA), a sesquiterpene plant hormone comprising fifteen carbon atoms, is one of the crucial endogenous hormones playing a central role in plant growth, development, and environmental responses. As one of the six classic plant hormones, ABA’s functions are extensive and diverse within the plant body, making it essential for understanding how plants interact with their environment. The discovery of ABA dates back to the 1960s when researchers first identified its role in maintaining plant dormancy and regulating growth [1,2]. The biosynthesis of ABA primarily occurs through the carotenoid pathway, beginning with the cleavage of carotenoids in the plastids. The key rate-limiting enzyme in this process is 9-cis-cyclocarotenoid dioxygenase (NCED), which is responsible for converting carotenoids into xanthoxin [3]. Xanthoxin is then oxidized in the cytoplasm to form ABA [3]. The metabolism of ABA is relatively simple and is primarily regulated through hydroxylation and glycosylation pathways, which modulate ABA levels in the plant to maintain dynamic equilibrium, adapting to different growth stages and environmental conditions (Figure 1A) [4,5,6].

The most extensively studied ABA receptors are the Pyrabatin resistance (PYR)/Pyrabatin resistance like (PYL)/Regulatory component of ABA receptors (RCAR) family (hereinafter referred to as PYL) [7,8,9]. By binding to ABA, they inhibit the activity of Protein phosphatase 2C (PP2C), thereby activating Sucrose Non-fermenting 1-related protein kinase 2 (SnRK2), which in turn initiates a cascade of downstream signaling, regulating gene expression (such as ABA-responsive element binding proteins (AREB)/ABA responsive cis-acting elements (ABRE)-binding factors (ABF) [10]), stomatal closure, and other physiological processes [11,12,13]. This precise regulatory mechanism enables ABA to effectively control various key developmental processes and environmental responses in plants [14,15,16,17]. During this process, ABA receptors employ a “Gate-Latch-Lock” mechanism for ABA-dependent regulation. PYLs contain an open ligand-binding pocket, where the “gate” is controlled by two highly conserved β-loops (serving as the “gate” and “lock”), which undergo conformational changes and close in response to ABA binding [18,19] (Figure 1B).

ABA plays a critical role in multiple stages of plant growth and development, including seed maturation, dormancy and germination, seedling development, stomatal movement, and the transition from vegetative to reproductive growth [20]. Additionally, ABA plays a significant role in helping plants cope with various environmental stresses such as drought, salinity, low temperatures, osmotic pressure, and mechanical injuries [21,22]. During seed development, ABA plays a decisive role by regulating seed dormancy and germination, enabling plants to initiate development under favorable environmental conditions [23,24]. ABA controls the accumulation of water and nutrients in seeds, as well as the activation of metabolic pathways, thereby modulating the speed of seed germination [25]. In the early stages of seed development, the accumulation of ABA promotes dormancy, while under suitable environmental conditions, the decrease in ABA levels triggers germination [26,27]. This process is crucial for plants to adapt to environmental changes and for enhancing seed survival rates. During fruit development, ABA works in coordination with other plant hormones to regulate processes such as sugar accumulation, color changes, and cell wall degradation [28]. Studies have shown that the dynamic changes in ABA levels throughout various stages of fruit development are closely associated with fruit maturation [29,30]. By modulating the ABA signaling pathway, the maturation period of fruits can be extended or shortened, thereby improving fruit quality and market value [31]. In recent years, the potential application of ABA signaling regulators in controlling seed and fruit development has garnered widespread attention. ABA signaling regulators, such as ABA receptor agonists or antagonists, can precisely modulate plant responses to ABA, influencing seed dormancy and germination, as well as fruit development and maturation [32]. In the field of breeding, a crucial strategy for improving seed quality and fruit development involves using marker-assisted selection or gene editing technologies to precisely regulate key genes in the ABA signaling pathway [33,34]. These technologies provide breeders with new tools to develop crop varieties with greater stress resistance and controlled developmental cycles, further advancing agricultural production.

Therefore, this paper systematically explores the critical role of ABA in seed development and fruit maturation. Unlike traditional models that view ABA as a single regulatory factor, we provide a detailed analysis of the complex interactions between ABA and other hormones, as well as their cooperative or antagonistic effects on seed development and fruit maturation. This cross-hormone interaction model offers a more comprehensive understanding of the dynamic balance of plant growth. In addition to summarizing the classical ABA regulatory model, we have expanded and supplemented it with the latest research findings, proposing new perspectives to offer researchers fresh insights into seed and fruit development. Furthermore, this review presents three agricultural application strategies based on ABA signal regulation, including the use of ABA analogs and ABA signal modulators (such as enhancers and inhibitors). We also discuss how precise control of key genes in the ABA signaling pathway, combined with molecular marker-assisted breeding and CRISPR-Cas9 gene editing, can enable precise regulation of seed development and fruit maturation. This refined, signal pathway-based breeding strategy breaks through traditional breeding models with enhanced specificity and practicality, providing an innovative and practical new approach to agricultural breeding that supports the application of research findings to real-world production. We emphasize the potential value of these strategies in agricultural production, offering a new reference for agricultural technology innovation.

## 2. From Seed Development to Dormancy Regulation

Fertilization in plants involves the fusion of male and female gametes to form a zygote, initiating the seed development process, which transforms the zygote into a mature, dry seed. The early stages of seed development involve the establishment of fundamental embryonic patterns, a phase often referred to as morphogenesis [35,36]. During the subsequent maturation phase, the zygote undergoes further development through cell division and differentiation. This includes the growth of the embryo, seed filling, the accumulation of nutrients, desiccation, and entry into dormancy [37]. In the final stages of seed development, the embryo dehydrates and adapts to dry conditions, entering a state of dormancy. Upon rehydration, the radicle breaks through the seed coat by elongating its cells, enabling germination; thus, the embryo transitions into the next generation of the plant [38].

### 2.1. Dynamics of ABA Levels During Seed Development

During seed development, ABA levels typically exhibit two peaks: the first occurs in the later stages of development, helping to halt embryonic cell division and promote nutrient accumulation; the second appears in the late maturation phase, which is crucial for inducing dormancy and facilitating the transition from maturation to germination [39,40]. Notably, the first ABA peak was initially observed in ABA-deficient tomato (*Solanum lycopersicum*) and maize (*Zea mays*) mutants [41]. Additionally, mutants lacking ABA or insensitive to ABA often exhibit premature germination and vivipary, further confirming the critical role of ABA in regulating seed dormancy and maturation [42]. 

In *Arabidopsis*, wheat (*Triticum aestivum*), and other crops, both maternal and embryonic tissues synthesize ABA. Maternal ABA plays a dominant role during the mid-stage of seed development, while embryonic ABA synthesis increases later in development [26,43,44,45]. ABA synthesized by the seed embryo is crucial for the induction and maintenance of dormancy [46]. Interestingly, the timing and levels of ABA synthesis vary significantly across different plants. Rice (*Oryza sativa*) seeds exhibit a single ABA peak during development, whereas wheat, barley (*Hordeum vulgare*), and other crops display two distinct peaks [47,48]. In certain barley varieties, higher ABA levels and increased sensitivity to ABA are observed, supporting its key role in reducing the risk of pre-harvest sprouting [49].

### 2.2. Regulation of Seed Development and Germination by the ABA Network

From a molecular perspective, 14 members of the PYL protein family of *Arabidopsis* have been shown to play significant roles in seed development, with the expression of 11 members undergoing notable changes during seed germination. PYL11 and PYL12 are specifically expressed in mature seeds, positively regulating ABA-mediated seed germination [50]. Although multiple mutations result in significant insensitivity to ABA, many single mutations do not cause an ABA-insensitive phenotype due to redundant functions [51]. For example, *Arabidopsis pyl* quadruple and decuple mutants exhibit impaired growth and an inability to produce seeds, while the duodecuple mutant shows severely impaired seed germination and seedling growth, with extreme insensitivity to ABA [52]. Additionally, the *pyl7/9* double mutant results in ABA-insensitive seed germination [53]. Interestingly, the *ospyl1/4/6* mutant in rice exhibits optimal growth under natural conditions and significantly increases grain yield while maintaining almost normal seed dormancy [54]. This finding provides new insights into the role of ABA in regulating seed development and yield.

**Figure 2:**
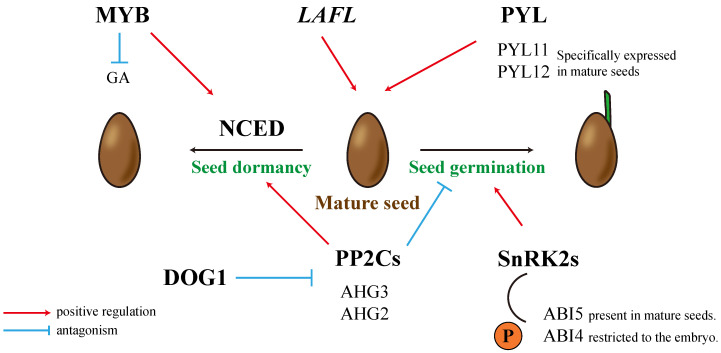
ABA signaling network and seed development dormancy. The *LAFL* gene group (*LEC1, ABI3, FUS3, and LEC2*) primarily functions in seed maturation, while PYL family members PYL11 and PYL12 are specifically expressed in mature seeds to facilitate ABA signaling. The key enzyme in ABA biosynthesis, NCED, promotes seed dormancy by increasing ABA accumulation. In contrast, PP2Cs (e.g., AHG3 and AHG2) and SnRK2s regulate sensitivity to ABA, with PP2Cs acting as negative regulators of seed germination. ABI5 and ABI4 are expressed in mature seeds and embryos, respectively, and play roles in the ABA signaling pathway. MYB inhibits GA biosynthesis, and since GA antagonizes ABA signaling, this interaction affects seed dormancy and germination. DOG1 regulates seed dormancy by inhibiting PP2Cs. In the figure, red arrows indicate positive regulation and blue lines indicate antagonistic interactions.

PP2Cs in the PYL-PP2Cs-SnRK2 complex act as negative regulators (Figure 2). Researchers compared the mutation effects of eight structurally related PP2C genes in Arabidopsis. It was found that *ABA-hypersensitive germination* (*AHG*) *3* is the most active PP2C gene in seeds, playing a central role in the ABA response [55]. Notably, the *atagh3* mutant does not exhibit any significant phenotype in adult plants [56]. *AGH1* is another *PP2C* gene that plays a crucial role in the seed′s response to ABA, and its mutant exhibits a strong ABA-insensitive phenotype [57]. *atagh1* plant seeds accumulate more ABA before stratification and exhibit increased seed dormancy, similar to the *atagh3* mutant with no noticeable phenotype in adulthood. Additionally, the *ahg1-1/3-1* double mutant is more sensitive than any single mutant, further indicating functional differences but partial overlap between AHG1 and AHG3 [58]. Researchers are particularly interested in the different sensitivities of AHG1 and AHG3 to inhibition by PYLs, especially the resistance of AHG1 to PYL receptor inhibition [59]. This suggests that this seed-specific phosphatase can regulate ABA signaling even in the presence of ABA and PYL receptors, thus controlling the active ABA signaling pathway during seed development.

Three SnRK2s (SnRK2.2, SnRK2.3, SnRK2.6) are found to act as positive regulators in many processes of seed development through ABA signal transduction [60,61]. Exogenous application of Nitric oxide (NO) can S-nitrosylate SnRK2.2 and SnRK2.3 to break seed dormancy and alleviate ABA′s inhibition of seed germination and early seedling growth [62]. The promoter of *ABA Insensitive* (*ABI*)*5* contains ABRE cis-acting elements, which can bind to and be activated by SnRKs, thereby regulating multiple ABA-related signaling pathways [63,64]. ABI5 is primarily expressed in mature seeds and is highly sensitive to ABA treatment, inhibiting seed germination and seedling establishment (Figure 2) [65]. Loss-of-function *abi5* mutants show significantly reduced sensitivity to ABA′s inhibition of seed germination, further confirming the critical role of ABI5 in regulating seed growth [66]. Evidence also shows that ABI5 interacts with ABI3 [67] and binds to the promoters of PYL11 and PYL12, further modulating ABA responses and forming a PYL–ABI5–ABA feedback mechanism, which finely regulates seed germination [50,68]. Research has shown that the Inducer of CBF expression (ICE) maintains appropriate ABA signaling levels during seed germination by antagonizing the activities of ABI5 and DELLA proteins [69]. Similarly, the brassinosteroid signaling pathway’s transcription factor Brinsensitive1-EMS-Suppressor 1 (BES1) interacts with ABI5, significantly inhibiting its binding to downstream gene promoters, thus promoting seed germination [68]. Additionally, *Arabidopsis* core circadian clock proteins (endogenous timing systems that synchronize internal biological processes with external environmental cycles, providing plants with adaptive advantages) collaborate with ABI5 to activate ABA responses during seed germination [70]. This offers new insights into how ABA signaling and the biological clock integrate through transcriptional complexes involving ABI5 and core circadian components. FCS-like zinc finger protein 13 (FLZ13) has also been identified as a new ABI5 interaction partner, working together to regulate seed germination [71].

ABI5 stability is equally important in seed germination and early growth. Brassinosteroid (BR) Insensitive 2 (BIN2) from the BR signaling pathway phosphorylates and stabilizes ABI5 to mediate ABA responses during seed germination [72]. In contrast, Ser/Thr protein phosphatase 6 (PP6) dephosphorylates and destabilizes ABI5 [73]. Similarly, the regulator of chromatin condensation 1 (RCC1) interacts directly with ABI5 at the phosphorylation site Ser-145, disrupting contact with SnRK2s and reducing ABI5 phosphorylation levels, negatively regulating ABI5 post-germination through a multidimensional mechanism in *Arabidopsis* [74]. The receptor for activated C kinase 1 (RACK1), a scaffolding protein, interacts with ABI5 through specific phosphorylation sites, inhibiting ABI5′s transcriptional activity and affecting protein stability, thus controlling seed germination and post-germination growth [75]. Additionally, post-translational modifications, such as small ubiquitin-like modifier (SUMO) E3 ligases binding with SUMO, negatively regulate ABA signaling through ABI5 [76], while NO stabilizes ABI5 by reducing ABA degradation during seed germination via S-nitrosylation [76]. 

ABI4 is a key transcription factor, primarily expressed in the embryo during seed maturation and early germination, where it inhibits lipid breakdown (Figure 2) [77,78]. The expression domain of the *ABI4* gene determines the sensitivity of lipid mobilization to ABA; thus, in the presence of ABA, ABI4 suppresses lipid mobilization to prevent seed germination [77]. Additionally, ABI4 can directly regulate the expression of target genes by binding to coupling element sequences in their promoters [78,79]. ABA also stabilizes ABI4 by binding to the *ABRE* elements in the promoters of *Cytochrome P450, Family 707, Subfamily A* (*CYP707A*) *1,* and *CYP707A2*, thereby increasing ABA levels and regulating primary seed dormancy [80,81]. ABI4 controls the expression of endogenous H_2_S-producing enzymes; the resulting H_2_S not only inhibits seed germination but also delays ABI4 degradation through sulfuration [82]. Moreover, the DELLA protein (an inhibitor of gibberellin acid (GA) signaling) specifically interacts with ABI4, forming a complex that mediates the antagonism between ABA and GA, thus ensuring normal plant development [83].

Studies suggest that ABI4 can form complexes with other transcription factors to co-regulate the seed germination process. For example, ABI4 participates in ABA and cytokinin signaling by suppressing A-type response regulators in *Arabidopsis* [84] or by binding to the promoter of *MUR4* encoding UDP-D-glucose 4-epimerase to positively regulate L-arabinose biosynthesis, thereby inhibiting seed germination [85]. ABI4 also interacts with phytochrome-interacting factor 4 (PIF4) to enhance ABA synthesis and signaling, promoting seed dormancy [86]. Under adverse conditions, ABI4 supports seed germination by regulating lipid mobilization through myeloblastosis transcription factor (MYB) 96 [87]. Additionally, evidence shows that ABI4 participates in reactive oxygen species (ROS) generation under stress to inhibit seed germination. For instance, under salt stress, ABI4 forms a regulatory module with respiratory burst oxidase homolog D (RbohD) and Vitamin C defective 2 (VTC2), reducing germination rates [88]. Consistently, under high-temperature stress, the release of ABI4 activity promotes RbohD expression, leading to an ROS burst that inhibits germination [89].

Another class of genes plays a crucial role in ABA-mediated development, particularly in seed maturation (Figure 2). These include the *LAFL* genes: *Leafy cotyledon 1* (*LEC1*), *ABI3*, *Fusca 3* (*FUS3*), and *Leafy cotyledon 2* (*LEC2*), as well as the AFL clade of B3 domain plant-specific transcription factors (AFL-B3), and the CCAAT-binding transcription factor (CBF) or nuclear factor Y (NF-Y) HAP3 subunit, LEC1, and LEC1-like (L1L) [45]. *LEC1* is essential for normal embryogenesis during both early and late stages, with its RNA accumulating specifically in embryonic cells and endosperm tissue during seed development, where it can induce embryonic development in vegetative cells [90]. *LEC2* directly controls the transcriptional programs involved in seed maturation [91]. A key feature of this gene family is the presence of both Sph/RY and *ABRE* motifs in their promoters, which are regulated by interactions between ABI3 and ABI5-related basic leucine zipper (bZIP) transcription factors. This coupling of the *LAFL* network with ABA signaling is mediated through the interaction of the N-terminal COAR (co-activator/co-repressor) domain of *ABI3* with *ABI5* and related bZIP factors [92,93]. ABRE motifs are also present in the promoters of other *LAFL* target genes, suggesting that other components of the *LAFL* network may also be co-regulated by ABA [94,95].

### 2.3. ABA Maintains Seed Dormancy

Dormancy is a temporary state of inactivity that is essential for preventing plant seeds from germinating under unfavorable conditions, helping to ensure species survival. During this stage, ABA helps align the germination process with favorable environmental conditions [45]. In cultivated crops, however, rapid and uniform germination is desired for higher yield and quality. Premature germination can lead to reduced yields and poorer grain quality, making it important to store seeds with an appropriate level of dormancy post-harvest [26]. Excessive dormancy, however, may result in uneven germination and unstable yields, making dormancy regulation critical for agricultural success. Seed development is complex, and significant progress has been made in understanding how ABA regulates seed maturation [96,97,98,99]. While many transcription factors controlling seed maturation have been identified, research on the transcriptional regulation of early embryogenesis remains limited.

ABA metabolism and signaling are key regulators of ABA-related seed dormancy, with endogenous ABA maintaining dormancy by inhibiting germination (Figure 2) [100]. Studies using ABA biosynthesis inhibitors have shown that de novo ABA synthesis plays a crucial role in sustaining seed dormancy. In *Arabidopsis*, ABA biosynthesis genes *NCED6* and *NCED9* are responsible for ABA accumulation during seed development and dormancy, and their mutants exhibit reduced ABA levels and dormancy in mature seeds [101]. Other ABA-deficient mutants, such as *aba1* and *aba2/3*, also show reduced dormancy [45]. *AtMYB96* induces primary dormancy by activating ABA biosynthesis genes *NCED2*, *NCED5*, *NCED6*, and *NCED9* while inhibiting GA biosynthesis genes [102]. *AtABI4* further enhances ABA biosynthesis by interacting with the promoter of *AtNCED6* and suppressing GA accumulation by regulating GA inactivation genes, strengthening seed dormancy [80,81]. Dormancy release involves CYP702A2, which catabolizes ABA in the embryo and endosperm [103].

Members of the *LAFL* gene family also play roles in dormancy acquisition. These genes control the arrest of embryo growth in mature seeds, and their mutants exhibit premature germination [104]. *Delay of germination1* (*DOG1*) is a key dormancy gene, with its mutation leading to complete dormancy release [105]. The amount of DOG1 protein in seeds determines the dormancy duration, and the protein loses its function during seed maturation [106]. Genetic studies suggest that DOG1 acts independently of ABA; however, both DOG1 and ABA are required to induce dormancy, and the absence of either results in dormancy defects, even if the other accumulates [106,107]. DOG1 controls dormancy by inhibiting specific PP2C phosphatases, including AHG1 and AHG2, which act downstream of DOG1 and represent convergence points for ABA and DOG1 pathways (Figure 2) [56]. Additionally, DOG1 is essential for other aspects of seed maturation, partly through interactions with components of the ABA signaling pathway [108].

In summary, the role of ABA in seed development extends beyond merely controlling seed dormancy; it is also crucial for overall seed development and maturation. A deeper understanding of ABA′s functions not only helps to uncover the fundamental mechanisms of plant development but also offers potential pathways through which to enhance agricultural productivity and crop stress resistance. As a central regulatory factor in seed development, the significance of ABA cannot be overstated.

## 3. ABA-Mediated Regulation of Fruit Development and Physiological Characteristics

Fruit development and maturation have always been a key focus in horticultural research, with ABA playing an important role in this process. Based on differences in respiration rates and ethylene (ETH) release patterns during ripening, fruits can be classified into climacteric (e.g., tomato, apple (*Malus domestica*), peach (*Prunus persica*)) and non-climacteric (e.g., strawberry (*Fragaria* spp.), grape (*Vitis vinifera*), citrus (*Citrus sinensis*)) types. Fruit development and maturation are complex biological processes involving a series of physiological, biochemical, and structural changes, such as alterations in color, texture, flavor, aroma, and nutrient content [109]. Although ETH plays a key role in fruit development and ripening, ABA also has a significant impact on these processes [29]. This chapter will focus on the role of ABA in this context. 

### 3.1. Regulation of ABA in Non-Climacteric Fruits

During the development of non-climacteric fruits, the accumulation of ABA significantly promotes fruit development, while a decrease in ABA levels leads to delayed development [110,111]. During seed formation and fruit enlargement, ABA reaches its peak through phloem transport, but in the later stages of fruit development, the output of ABA through the phloem decreases, resulting in its accumulation in the fruit [29].

The synthesis, metabolism, transport, and signaling of ABA largely regulate its content in fruit cells. For example, altering the expression of the key metabolic enzyme FveCYP707A4a in wild strawberries (*Fragaria vesca*) can influence endogenous ABA levels and affect the expression of *FveNCED* [112]. Feedback and feedforward loops tightly connect the synthesis and degradation of ABA, limiting its content during fruit development but rapidly increasing its levels at the onset of the process. Similarly, in blueberries (*Vaccinium myrtillus*), ABA accumulates significantly during fruit development, with the expression of *VmNCED1* increasing accordingly. Exogenous ABA treatments promote the expression of development-related genes in blueberries, accelerating processes like fruit softening, which highlights the key role of ABA in blueberry fruit development [113]. In citrus, ABA can limit its own accumulation on the 14th day after exogenous ABA application by inducing the expression of ABA 8′-hydroxylase 1 and reducing the expression of *CsNCED1* [114]. In cucumbers (*Cucumis sativus*), exogenous ABA applied at the turning stage promotes fruit development, with ABA levels peaking in the flesh before full maturity [115]. It is generally believed that the development of grapes is primarily regulated by ABA, and its interaction with ETH may be essential for initiating berry development [116]. When ABA levels peak, a portion of it is stored in the form of ABA-GE. After harvest, abiotic stresses such as dehydration or harvest shock can induce the transcription of *VvNCED1* and promote the accumulation of ABA, thereby triggering the fruits′ aging process [116]. 

During fruit development, post-translational modifications play a crucial role in fine-tuning key components of the ABA signaling pathway, ensuring optimal fruit quality and timing [30]. Phosphorylation has been shown to influence the dynamic changes in ABA signaling proteins during the development of peppers [117]. Research has also revealed the role of m^6^A modification in stabilizing *NCED5* and *AREB1* mRNAs, promoting ABA biosynthesis and signaling, and thereby accelerating strawberry fruit development [118]. In grapes, ABA treatments have been shown to alter overall DNA methylation levels, inducing changes in genes related to development and stress responses. This provides new insights into the epigenetic regulation of non-climacteric fruit development by ABA [119].

### 3.2. Regulation of ABA and Ethylene in the Development of Climacteric Fruits

ETH was the first substance identified as promoting development in climacteric fruits [120]. The fruit development process requires high levels of ETH production and frequent respiration, suggesting a possible synergistic effect between ABA and ETH, with their interaction jointly regulating fruit development [121]. The accumulation of ABA occurs before ETH production, indicating that ABA may function as an upstream regulator of ETH biosynthesis, suppressing ETH synthesis until appropriate hormone levels are reached. Once this threshold is met, ABA promotes the release of ETH by inducing the expression of ETH biosynthesis genes *1-aminocyclopropane-1-carboxylic acid oxidase* (*ACO*) and *1-aminocyclopropane-1-carboxylic acid synthase* (*ACS*), accelerating further fruit development [122].

Tomato, as a model for climacteric fruits, demonstrates that exogenous ABA can accelerate post-harvest development and influence metabolic regulation. Studies have shown that endogenous ABA accumulates before ETH production, and ABA treatment advances the peaks of both ETH and ABA expression. However, exogenous ETH treatment only affects the ETH peak, with minimal impact on ABA levels [123]. These findings suggest that endogenous ABA plays a crucial role in the early stages of fruit development, while ETH′s role is more prominent in the later stages. ABA likely acts as an upstream regulator, controlling ETH synthesis and signaling in the ABA-ETH regulatory network, where ABA positively influences ETH, and ETH may serve as a “hub” in the process. Similarly, in mango (*Mangifera indica*), exogenous ABA treatment promotes the activity and accumulation of key ETH synthesis enzymes ACO and ACS, accelerating ETH production. Conversely, the use of ABA inhibitors significantly delays or suppresses ETH activity in the fruit flesh [124].

Additionally, researchers have proposed a new model for ABA–ETH regulation of fruit development, known as the NAC (No apical meristem, ATAF1/2, and Cup-shaped cotyledon)–ABA–ETH regulatory model [125]. In this model, NAC transcription factors play a key regulatory role during fruit development by modulating ABA-induced ETH production, thereby indirectly controlling the initiation of ETH synthesis and the fruit development process [126]. Among them, SNAC4 (a member of the NAC transcription factor family) acts as a convergence point for ABA and ETH signals within this regulatory network, playing a dual regulatory role. SNAC4 not only directly regulates the expression of key ETH biosynthesis genes such as *SlACS2* and *SlACO1* [127] but also influences ABA biosynthesis and metabolism by regulating ABA synthesis genes like *SlNCED1* and *SlNCED2* [128]. SNAC9, on the other hand, is primarily responsible for sensing and transmitting ABA signals. It interacts directly with the ABA receptor *SlPYL9* and activates downstream genes in the ABA signaling pathway, such as *SAPK3* and *SlAREB1* [126] The cooperation between SNAC4 and SNAC9 forms a complex regulatory network, where SNAC9 mediates ABA signal perception to regulate the early stages of fruit development, while SNAC4 simultaneously regulates both ABA and ETH ensuring balanced fruit development. NOR, another member of the NAC domain family, has been shown to act upstream of ETH. Like SlAREB1, NOR expression is induced by ABA, with *SlAREB1* transcription peaking before *NOR* during fruit ripening. *NOR* is a direct target, indicating that *SlAREB1* mediates ABA signaling to activate *NOR* transcription and ultimately promote ETH synthesis [129].

Although ABA and ETH are generally considered to act synergistically, in some cases, ABA may inhibit ETH synthesis, and ETH may, in turn, antagonize the effects of ABA. This relationship can vary depending on the type of fruit or the developmental stage [111]. For example, the inhibition of the key ABA biosynthesis gene *NCED1* can lead to an increase in ETH synthesis, further demonstrating that ABA may, in certain circumstances, delay fruit development by suppressing ETH activity [130]. Additionally, the ethylene response factor (ERF) family may regulate fruit development by modulating ABA biosynthesis genes during fruit development [131]. For instance, *PpeERF2* binds to the promoters of two key ABA biosynthesis genes (*PpeNCED2* and *PpeNCED3*) and regulates fruit maturation in peaches by inhibiting their transcription. Additionally, several ETH-responsive elements have been identified in the promoters of *PpeNCED2* and *PpeNCED3* [131]. This suggests that ETH may influence fruit development under certain conditions by suppressing ABA activity.

In summary, the ABA-mediated mechanism of ETH production during fruit ripening has not yet been thoroughly studied. However, ABA′s precise regulation of ETH synthesis is crucial for the development of climacteric fruits. Therefore, further research is needed to determine whether ABA promotes or inhibits fruit development at different stages of maturation.

### 3.3. Regulatory Role in Fruit Quality and Appearance Characteristics

**Figure 3:**
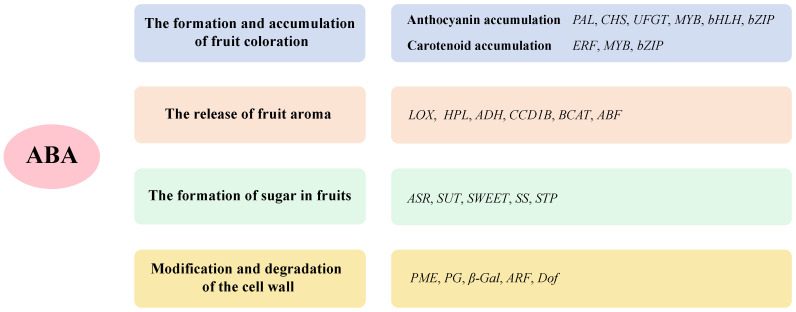
Transcriptional regulation of fruit quality and appearance traits by ABA. ABA plays a crucial role in the transcriptional regulation of fruit quality and appearance traits, affecting various aspects such as color, texture, nutrient composition, and fruit firmness.

The color of a fruit is a key indicator of its physiological maturity and quality (Figure 3). Early studies demonstrated that exogenous ABA application promotes fruit coloration, as observed in grapefruit, a finding later confirmed in other fruits [111]. In *FaNCED1-RNAi* strawberries, reduced ABA levels resulted in a colorless phenotype, which was restored by exogenous ABA, indicating ABA′s crucial role in regulating fruit coloration with direct implications for agricultural production and market value [132]. Anthocyanins and carotenoids, two important plant pigments, contribute not only to fruit coloration but also to various biological functions, providing significant nutritional and health benefits [133].

During ripening, fruit color changes due to the accumulation of pigments, and ABA promotes anthocyanin accumulation, enhancing coloration [134]. In grapes, the overexpression of *VvPYL1* increased anthocyanin accumulation and induced the transcription of ABA-responsive genes [135]. Anthocyanin biosynthesis involves structural genes [136] like *Phenylalanine ammonia*-*lyase* (*PAL*), *Chalcone Synthase* (*CHS*), and *Flavonoid 3-O-glucosyltransferase* (*UFGT*), and regulatory genes, including transcription factors from the MYB, basic Helix-Loop-Helix (bHLH), bZIP, and WD40 families [137,138]. For example, in apples, ABA induces *MdbZIP44*, which binds to *MdMYB1*, promoting anthocyanin accumulation [139]. Similarly, in tomatoes, *SlAREB1* controls anthocyanin biosynthesis under cold conditions via an ABA-dependent pathway [140]. In blueberries, *VmbZIP55* responds to ABA signals by interacting with the *G-Box* motif on the *VmMYB1* promoter, activating its expression and promoting anthocyanin synthesis [141].

Sucrose and ABA both play significant roles in enhancing fruit coloration and anthocyanin content (Figure 3). When sucrose supply is low, increased ABA levels can promote anthocyanin production, as demonstrated in grape berries, where ABA’s effects are mainly mediated through the upregulation of transcription factors in the phenylpropanoid pathway [142]. ABA treatment in grapes increases the expression of the key anthocyanin synthesis enzyme UFGT and boosts the production of various anthocyanins during ripening [143]. In the non-climacteric fruit ripening model, both sugars and ABA are considered central regulatory factors in anthocyanin biosynthesis. However, research on blueberries shows that ABA treatment upregulates anthocyanin-related biosynthesis genes, while *VmNCED1-RNAi* leads to a downregulation of these genes, indicating that ABA is a crucial positive regulator in blueberry fruit ripening. Conversely, sugars (glucose, fructose, and sucrose) play a lesser role in regulating blueberry ripening, as they do not induce anthocyanin or ABA biosynthesis in blueberry fruits [115]. These findings suggest that different fruits may have varying mechanisms for regulating anthocyanin biosynthesis and further research is warranted to explore these differences.

ABA also plays a significant role in carotenoid accumulation (Figure 3). Exogenous ABA can increase carotenoid content in fruits like citrus and tomatoes [144]. ABA-deficient mutants lead to an increase in carotenoid accumulation in the fruit [145]. In citrus, transcription factors CsERF110 and CsERF53 activate carotenoid metabolism genes to enhance carotenoid accumulation [146], while CsbZIP44 regulates ABA-mediated carotenoid biosynthesis by directly binding to promoters of carotenoid metabolism genes [147]. In apples, *MdMYBS1* is closely linked to both carotenoid and ABA content, promoting β-carotene synthesis and ABA accumulation [148]. Additionally, ABA 8’-hydroxylase, a key regulator of endogenous ABA homeostasis, also plays a crucial role in carotenoid accumulation. Silencing this enzyme in sweet cherries (*Prunus avium*) increases ABA levels, thereby accelerating fruit coloration and ripening [149]. In cucumbers, fruit flesh color is regulated by modulating carotenoid biosynthesis, influencing the yellow pigmentation [33].

Fruit aroma is derived from various volatile compounds, and ABA affects the expression of these volatiles in fruits (Figure 3). Exogenous ABA accelerates the production of short-chain ester aroma compounds and the expression of biosynthetic genes during apple ripening [150], while ABA biosynthesis inhibitors downregulate volatile compound pathways [151]. ABA regulates the expression of volatile biosynthesis genes such as *Lipoxygenase* (*LOX*), *Hydroperoxide lyase* (*HPL*), *Alcohol dehydrogenase* (*ADH*), *Carotenoid cleavage dioxygenase 1B* (*CCD1B*), and *Branched-chain amino acid transaminase* (*BCAT*) [152]. In cherry tomatoes (*Solanum lycopersicum*), exogenous ABA increases volatile compound accumulation and induces key biosynthetic gene expression. Promoter analysis revealed that eight out of twelve genes involved in volatile biosynthesis contain *ABRE* motifs, suggesting ABA’s role in modulating gene expression to influence volatile release [152]. In apples, the ABA-responsive transcription factor MdABF2 directly promotes the transcription of volatile biosynthesis genes. Its overexpression enhances volatile compound production, effects that are amplified by ABA treatment [153].

Fruit sugar content, a key indicator of quality, also influences flavor and market value. Recent studies have shown that ABA dynamically regulates fruit sugar content by impacting sugar metabolism, transport, and accumulation [154] (Figure 3). ABA regulates the expression and activity of sugar-metabolizing enzymes, promoting sugar synthesis and accumulation. Additionally, ABA influences sugar transport proteins, optimizing sugar distribution and enhancing fruit sweetness [154]. The *ABA stress ripening* (*ASR*) gene is a cross-signaling factor between ABA and sucrose, with its expression influenced by both [155]. Under ABA treatment, the transcription factor AREB activates starch synthase and sugar transporter genes like *Sucrose transporter 2* (*SUT2*), promoting soluble sugar accumulation [156]. Furthermore, transcription factors such as bZIP23 and bZIP46 regulate sugar transporter proteins like *Sugar will eventually be exported transporters 9b* (*SWEET9b*), impacting sugar accumulation [157]. ABA also influences sugar accumulation through Sucrose synthase3 (SS3) [158], or Sugar transport protein13 (STP13), and the *Sugar Phosphate/Phosphate Translocator (SPT)* module [159]. These studies highlight ABA′s role in sugar regulation during fruit maturation, with additional effects on hexose transport proteins like Hexose transporter (HT) [160]. Moreover, ABA plays a significant role in regulating key enzymes involved in fructan and sucrose metabolism in wheat [161].

ABA significantly influences fruit softening by regulating cell wall modification and degradation pathways, thereby altering fruit texture and commercial value (Figure 3). Exogenous ABA treatment in blueberries accelerates softening, increases soluble pectin content, and reduces cellulose and hemicellulose levels [162]. Additionally, ABA activates enzymes such as Pectin methylesterase (PME), Polygalacturonase (PG), and β-galactosidase (β-Gal), enhancing cell wall metabolism [163]. For example, in melons (*Cucumis melo*), ABA promotes ripening and softening by up-regulating ETH synthesis-related genes and the cell wall-degrading enzyme PG1 [164]. Through inhibition of Auxin response factor 8 (ARF8), ABA activates DNA binding with one finger (Dof) 2/15 transcription, which binds directly to the promoter regions of cell wall-modifying enzymes, forming the ABA-PavARF8-PavDofs feedback loop to promote cell wall softening [165]. Additionally, heterologous expression of *CsABI5-Like* in citrus binds to the tomato *SlPL* promoter, reducing fruit hardness in transgenic plants [166].

ABA is a key hormone in fruit development, playing both direct and indirect roles in regulating various physiological functions related to fruit growth. Research has primarily focused on climacteric fruits such as tomatoes, apples, and bananas, while studies on non-climacteric fruits have progressed more slowly. This is mainly due to the low efficiency of genetic transformation and long fruiting cycles in crops like citrus and grapes, which limits detailed analysis of their molecular mechanisms during fruit development. Additionally, while the regulatory roles of individual plant hormones are relatively well understood, the complex interactions among multiple hormones remain unclear. Further exploration of these synergistic and antagonistic hormonal interactions will be an important direction and challenge for future research. 

## 4. Strategies Based on ABA Signaling in Agronomic Production

ABA plays a significant role in plant growth and development, especially during seed development and fruit ripening stages. With advances in our understanding of ABA signaling pathways, the potential applications of ABA signaling modulators in agriculture have garnered wide attention. ABA analogs, receptor agonists, and antagonists can precisely regulate ABA signal transduction, enabling accurate control over seed development and fruit maturation [167]. Additionally, molecular breeding strategies based on ABA signaling show great promise. By enhancing ABA-related genes or modulating their expression, it is possible to develop crop varieties with improved stress tolerance, higher yields, and superior quality [39]. These advancements offer new approaches to address global climate change and resource constraints in agriculture while also laying a solid foundation for achieving sustainable agricultural goals.

### 4.1. ABA and Its Analogs

ABA plays a crucial role in regulating various physiological processes during seed development and fruit ripening. In agriculture, ABA treatments are commonly used to adjust seed physiological states, improve germination, and enhance adaptability. These treatments can be applied during seed storage, pre-sowing, and seed production [167]. Exogenous ABA can inhibit embryo development in recalcitrant seeds, promote dormancy, and delay germination. Studies show that ABA-regulated hormone pathways and Mitogen-activated protein kinase (MAPK) signaling pathways are significantly enriched in differentially expressed genes [168]. Under drought stress, ABA treatment improves maize seed germination rate, vigor, and seedling biomass, while increasing endogenous ABA content to enhance drought adaptation [169]. In soybean (*Glycine max*), exogenous auxin suppresses germination by enhancing ABA biosynthesis and inhibiting GA biosynthesis, lowering the GA/ABA ratio. ABA biosynthesis inhibitors reverse auxin-induced germination delays, while GA inhibitors suppress germination [170].

ABA is also used to improve fruit quality by increasing sugar content and nutrients. In tomatoes, ABA increases soluble sugars and decreases organic acids, improving fruit quality [171]. ABA accelerates citrus fruit coloration and reduces organic acid content [114]. During sweet cherry maturation, ABA treatment strengthens the cell wall and alters cuticle composition, enhancing fruit quality [172]. Post-harvest, ABA increases soluble sugars and prolongs strawberry shelf life by suppressing softening enzymes like PG and Pectate lyase (PL), improving cell adhesion [31]. In cassava (*Manihot esculenta*), ABA treatment reduces H_2_O_2_ content and extends shelf life by raising endogenous ABA levels [173].

Despite these benefits, ABA′s instability under ultraviolet light and rapid degradation limit its efficacy in field trials [174]. In contrast, ABA analogs offer better stability and effectiveness. For instance, 2′,3′-benzo-iso-ABA is a potent ABA analog with higher ABA-like activity, showing stronger inhibitory effects on seed germination and seedling elongation in various crops (Table 1) [175]. Photostability issues arise due to ABA’s side chain structure, but chemical modifications, such as substituting the diene acid on the side chain, improve stability while retaining biological activity [176]. The (+)-BP2A series compounds address ABA′s photostability by replacing the diene acid with phenylacetic acid, showing high activity in inhibiting seed germination in multiple species (Table 1) [177]. Other analogs, like (+)-tetralone ABA, modify the cyclohexenone ring to enhance persistence and bioactivity (Table 1) [178]. Additionally, replacing the cyclohexenone ring with a cyanocyclopropyl group increases transpiration inhibition, further extending its biological effects [179].

### 4.2. ABA Signaling Regulators

Natural ABA has a complex structure, resulting in high production costs and challenges for large-scale industrial synthesis. Consequently, scientists are focusing on developing small molecules with simpler structures and targeted ABA receptor activity. ABA receptor agonists mimic ABA′s action, allowing precise regulation of key processes like seed germination, seedling growth, and fruit maturation [167]. These agonists can delay germination, ensuring seeds remain dormant under unfavorable conditions, which improves germination rates and seedling survival. In fruit development, they regulate maturation speed and quality, helping to control harvest timing and enhance fruit quality [167].

Quinabactin is a well-known ABA receptor agonist that inhibits tomato seed germination (Table 1) [181]. Another agonist containing a phthalimide structure forms hydrophobic interactions with ABA receptor residues, showing inhibitory effects on seed germination [187]. New Opabactin analogs, designed through molecular docking, also inhibit seed germination in *Arabidopsis* and rice (Table 1) [182]. Additionally, (Julolidine and fluorine containing ABA receptor activator) JFA 1 and JFA2 were identified as ABA receptor agonists using a wheat cell-free screening system, inhibiting seed germination by activating PYR1 and PYL1 receptors (Table 1) [183]. These findings highlight the potential of ABA receptor agonists in optimizing plant development and improving agricultural outcomes. Overall, ABA receptor agonists significantly impact plant developmental processes by precisely modulating ABA signaling within the plant. Their agricultural applications hold substantial promise, particularly in optimizing seed germination and fruit maturation.

ABA receptor antagonists block ABA′s binding to receptors, thus suppressing ABA signaling pathways [167]. These compounds have been used primarily in research but show promise for agricultural applications. Early structural studies using X-ray crystallography revealed a channel above the 3′ ring -CH of ABA, which opens at the PP2C binding interface. ABA analogs with sufficiently long 3’ alkyl chains are predicted to traverse this channel and block the PYL-PP2C interaction [188]. Using this conformational constraint method, the antagonists (+)-PAT3 and (+)-PATT1 were designed, and they exhibit stronger inhibitory effects on ABA-induced seed germination in *Arabidopsis* compared to the original antagonists (Table 1) [184]. Chemical genetics screening led to the identification of ABA Antagonist1 (AA1), the first broad-spectrum ABA receptor antagonist in Arabidopsis, with sufficient activity to block ABA signaling (Table 1) [185]. Additionally, a derivative library was created using click chemistry, optimizing into the broad-spectrum receptor antagonist Aantabatin. This compound exhibited higher activity in vivo, accelerating seed germination in *Arabidopsis*, tomato, and barley, indicating that it can serve as a germination stimulant by limiting the regulation of seed germination by endogenous ABA signaling (Table 1) [186].

ABA signaling regulators hold great potential for agricultural applications, yet large-scale use still faces challenges related to stability and precise modulation. Future research will focus on developing more specific and controllable ABA regulators and exploring synergies with other plant hormone regulators to optimize crop growth performance and quality.

### 4.3. Insights from ABA Signaling for Breeding

Seed germination is a crucial stage in the crop life cycle, regulated by various internal and external factors, directly impacting planting density, germination rate, and early growth vigor. Among these factors, ABA serves as a primary inhibitor, playing a significant role, particularly in controlling seed dormancy. In barley, the ABA/GA ratio determines the genetic variation in seed dormancy. Changes in ABA levels are regulated by the expression of genes such as *NCED1* and/or *CYP707A*s, while GA levels are modulated by GA 20-oxidase and 3-oxidase expression. These genes are potential targets for developing molecular tools to enhance pre-harvest sprouting resistance in barley, allowing for precise control over seed dormancy and germination timing [99]. ABA levels during seed germination directly affect the physiological state of the seeds. Targeted mutations in specific sites of the PYR/PYL receptor family can create crop varieties with enhanced stress resistance, suitable for cultivation under harsh environmental conditions. For example, the creation of a soybean *gmpyl* gene knockout mutant resulted in increased plant height and branching compared to wild types. Under ABA stress, certain positive regulators of germination were activated, promoting seed germination [189]. Similarly, introducing an ABA biosynthesis gene *NCED* driven by an ABA-responsive promoter into common wheat resulted in ABA accumulation in the embryo, enhancing grain dormancy and delaying germination by several days [190]. Moreover, editing key transcription factors can produce crop varieties that are less sensitive to ABA, resulting in higher germination rates and stable seedling establishment under adverse conditions. ABI5, a key component in regulating plant growth arrest, shows heightened sensitivity to ABA when overexpressed, whereas *abi5* mutants exhibit insensitivity to ABA [191]. The *Medicago truncatula mtabi4* mutant displays seeds with increased chlorophyll and reduced dormancy, enabling seed development in darkness similar to wild types [192]. In tomatoes, overexpression of SlABI3 activates the downstream gene *SlABI5*, conferring hypersensitivity to exogenous ABA during seed germination and primary root growth [193]. By screening or designing germplasm to regulate the key signaling elements, precise control over seed germination timing can be achieved, which is crucial for developing crops suited to various climatic conditions and farming practices.

ABA plays a crucial synergistic role in fruit development by regulating gene expression, affecting processes such as sugar accumulation, color changes, and cell wall degradation. In breeding strategies, precisely regulating the intensity and timing of ABA signaling can allow for more accurate control of fruit development processes. For commercial fruit tree varieties, extending the harvest period or delaying development is one of the key breeding goals. By screening or engineering key genes in the ABA signaling pathway, breeders can develop new varieties with controlled development periods and improved storage qualities. For example, RNA interference constructs driven by a fruit-specific E8 promoter significantly reduced NCED activity, resulting in decreased expression of genes encoding major cell wall-degrading enzymes, leading to a notable extension of tomato shelf life to 15–29 days compared to 7 days for control fruits, with a 30–45% increase in fruit firmness at maturity [194]. Additionally, overexpression of the β-glucosidase gene *BG1* in tomatoes increased ABA levels prior to fruit ripening, advancing the ripening time by 3–4 days and inducing earlier ETH release compared to wild-type fruits [195].

During the ABA-regulated processes of seed germination and fruit development, multiple key genes play crucial roles. These genes can be effectively tracked and utilized through marker-assisted selection. Research indicates a strong correlation between genetic variation in seed dormancy in wheat and ABA levels, which are closely associated with the expression of ABA biosynthesis genes *TaNCED1* and *TaNCED2*. There is also a high positive correlation between the expression patterns of *TaABI5* and *TaNCED1* or *TaNCED2* [98]. This highlights the potential use of these genes in developing molecular markers for pre-harvest sprouting resistance and seed dormancy in wheat. Furthermore, transgenic technology or gene editing methods can directly modify ABA signaling pathways. In pear (*Pyrus communis*) breeding, the goal of achieving smaller and compact traits was achieved through Agrobacterium-mediated transformation overexpressing the ABA-related S-acyltransferase gene *PbPAT14*, resulting in a dwarf phenotype [196]. CRISPR/Cas9, a revolutionary gene editing technology, allows for the precise modification of DNA and has become a core tool in genetic engineering. CRISPR/Cas9-based editing of ABA-related gene expression is suitable for rapid breeding in agricultural production [197]. For example, CRISPR/Cas9-induced mutations in *OsNCED3* in rice resulted in reduced ABA levels and increased GA levels in embryos, promoting embryo growth and breaking seed dormancy prior to harvest, thereby enhancing pre-harvest germination [198]. Similarly, targeting and editing three genes encoding 8’-hydroxylase in rice with CRISPR/Cas9 significantly increased seed dormancy without affecting yield [199].

As research into plant hormone biology advances, ABA signaling-based breeding strategies will play an increasingly important role in global agricultural production. By precisely regulating ABA signaling pathways, future breeding efforts are expected to improve crop adaptability, nutritional value, and economic benefits, contributing significantly to global food security and sustainable agricultural production.

## 5. Future Perspectives and Outlook

In the context of global climate change, resource constraints, and population growth, agriculture faces unprecedented challenges. To meet the increasing food demand while ensuring environmental sustainability, the development and application of novel agricultural technologies are crucial. In this process, plant hormone regulation, particularly the role of ABA in seed and fruit development, is gradually becoming an important tool for enhancing crop yield and quality. Future research into ABA signaling pathways and their application in agricultural production will drive innovations in crop breeding and agronomic strategies, helping the agriculture industry address multiple challenges.

With the rapid advancement of molecular biology technologies, future research will further uncover the complex regulatory mechanisms of ABA signaling pathways. Modern gene-editing tools, such as CRISPR-Cas9, allow for precise adjustment of ABA-related gene expression, controlling key developmental processes like seed germination, seedling growth, and fruit development. This technology has potential applications not only in laboratory settings but also in practical agricultural production. Future research directions may include developing targeted gene-editing strategies based on ABA signaling pathways, enabling researchers to flexibly adjust crop growth cycles according to climate conditions or market demands. These technological advancements will facilitate precision and personalized management in agriculture, enhancing resource utilization efficiency, reducing chemical inputs, and increasing the added value of agricultural products [200].

The innovation and application of ABA receptor modulators will be a key area in the future of agricultural technology. As our understanding of ABA signaling deepens, researchers are focused on designing more efficient ABA receptor modulators for precise control of plant physiological processes. Future modulators will be more specific, acting precisely at certain developmental stages or under environmental stress. By optimizing molecular structures, these modulators can bind specifically to ABA receptors, reducing side effects and enabling refined physiological control. Additionally, new compounds will be designed to better withstand environmental changes, ensuring durability and effectiveness in field applications [201]. In practice, ABA receptor modulators hold significant potential not only for improving crop stress resistance but also for regulating plant growth, controlling crop maturation timing, and enhancing product quality. Integrating these modulators into modern agricultural management systems will enable farmers to better adapt to climate change and market demands, optimizing crop performance.

As an important signaling molecule for plants to cope with environmental stress, ABA’s role in stress resistance has seen significant progress [202]. Future research will delve deeper into the mechanisms of ABA under various stress conditions, especially in the context of multiple stress responses. Combining environmental sensing technologies with precise ABA signaling regulation strategies will enhance agricultural production’s ability to handle the uncertainties brought by climate change. For example, with the intensification of global climate change, crops will face more frequent and severe drought, high temperatures, and salinity stresses. Developing and applying agronomic strategies to regulate ABA signaling can help crops maintain high yield and quality under these extreme conditions. Additionally, these strategies will contribute to reducing reliance on irrigation water, improving water resource utilization efficiency, and promoting sustainable agriculture [203].

One trend in future agriculture is the widespread adoption and application of smart agriculture technologies. By integrating sensor technology, big data analysis, and machine learning with ABA signaling regulation, agriculture can achieve real-time monitoring and dynamic management. Real-time monitoring of soil moisture and temperature, combined with the precise application of ABA signaling modulators, allows farmers to regulate crop growth conditions at optimal times, reducing resource waste and improving crop yield and quality [204]. Additionally, smart agriculture technologies can help farmers optimize irrigation and fertilization plans, reducing the use of agricultural inputs and lowering production costs and environmental impacts. With automated and intelligent management systems, the application of ABA signaling regulation in agriculture will become more precise and efficient, advancing it toward modernization and sustainability.

Therefore, the application of ABA signaling regulation technology in agricultural production has broad prospects. Future research and practice will further drive development in this field. Through continued innovation and multi-party collaboration, we hope to achieve more efficient, environmentally friendly, and sustainable agricultural production systems in the near future, making a positive contribution to global food security and environmental protection.

## Figures and Tables

**Table 1:** Chemical structures, design strategies, and functions of ABA signaling modulators.

Category	Designation	Chemical Structure	Design Strategy	Function	References
ABA analogs	2′,3′-iso-PhABA	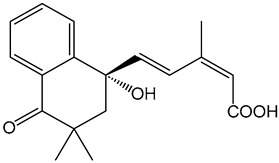	The introduction of a benzo ring at the 2′, 3′ position alters the molecular conformation.	Inhibitory effects on lettuce (*Lactuca sativa*) and *Arabidopsis* seed germination, wheat germination, and rice seedling elongation.	[175]
	(+)-BP2A	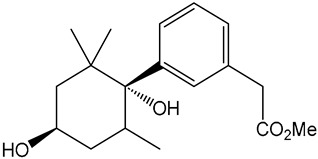	The diene on the side chain of ABA is replaced by a phenylacetic acid group.	ABA-like activity that inhibits the germination of tomato, lettuce, and rice seeds.	[177]
	9′,9′-difluoro-ABA	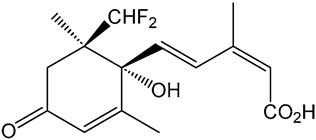	Chemical modifications to stabilize the cyclohexanone ring.	In radish seedlings and rice seedlings, the deactivation rate of ABA analogs is slower than that of ABA.	[180]
	(+)-tetralone ABA	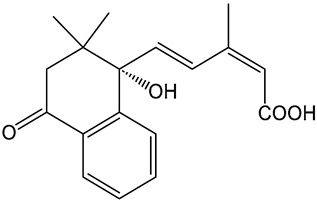	Fusion of a cyclohexenone ring with a benzene ring.	More effectively complements the growth retardation of ABA-deficient *Arabidopsis* mutants than natural ABA.	[178]
ABA agonists	Quinabactin	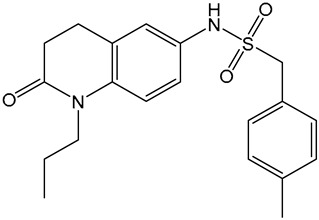	Hydrogen bonds or halogen bonds form hydrophobic interactions with amino acid residues of the ABA receptor.	An inhibitory effect on seed germination.	[181]
	Opabactin	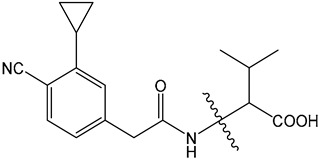	Structure-based design and molecular docking screening.	Inhibits seed germination and seedling growth in *Arabidopsis* and rice.	[182]
	JFA	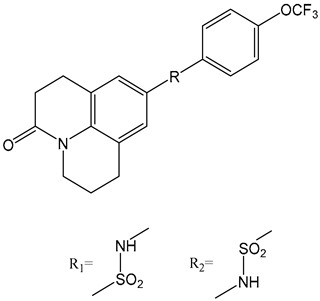	Cell-free drug screening system for wheat.	Activation of PYR1 and PYL1 inhibits seed germination and cotyledon greening in seedlings.	[183]
ABA antagonists	(+)-PAT	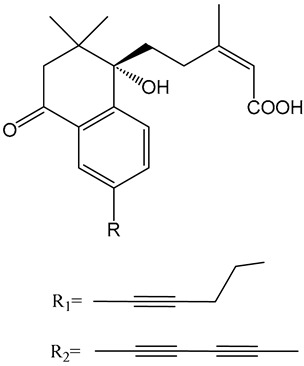	Conformational constraint method.	Inhibits ABA-induced seed germination in *Arabidopsis*.	[184]
	AAT1	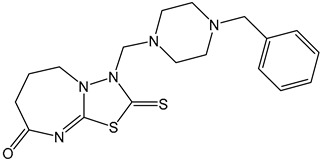	Chemical Genetics Screening	Broad-spectrum *Arabidopsis* ABA receptor antagonist.	[185]
	Aantabatin	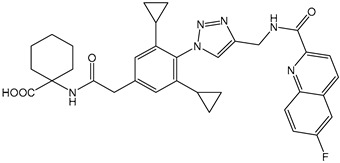	Click chemistry for creating a derivative library.	Accelerates seed germination in *Arabidopsis*, tomato, and barley.	[186]

## Abbreviations

Abbreviation	Full term
ABA	Abscisic acid
ABA-GE	ABA-glucosyl ester
ABF	ABRE-binding factors
ABI	ABA Insensitive
ABRE	ABA responsive cis-acting elements
ACO	1-aminocyclopropane-1-carboxylic acid oxidase
ACS	1-aminocyclopropane-1-carboxylic acid synthase
AHG	ABA-hypersensitive germination
AREB	ABA-responsive element binding protein
bZIP	basic leucine zipper
CYP707A	Cytochrome P450, Family 707, Subfamily A
DOG1	Delay of germination 1
ERF	Ethylene response factors
ETH	Ethylene
GA	Gibberellin acid
MYB	Myeloblastosis transcription factors
NAC	No apical meristem, ATAF1/2, and Cup-shaped cotyledon
NCBD	9-cis-cyclocarotenoid dioxygenase
PG	polygalacturonase
PP2C	Protein phosphatase 2C
PYL	Pyrabatin resistance like
PYR	Pyrabatin resistance
RCAR	Regularly component of ABA receptors
ROS	Reactive oxygen species
SnRK2	Sucrose Non-fermenting 1 (SNF1)-related protein kinase 2
UGT	ABA-uridine diphosphate (UDP) glucosyltransferase
